# Estimating malaria transmission intensity from *Plasmodium falciparum* serological data using antibody density models

**DOI:** 10.1186/s12936-016-1121-0

**Published:** 2016-02-09

**Authors:** Emilie Pothin, Neil M. Ferguson, Chris J. Drakeley, Azra C. Ghani

**Affiliations:** Department of Infectious Disease Epidemiology, MRC Centre for Outbreak Analysis and Modelling, Imperial College London, London, UK; Department of Epidemiology and Public Health, Swiss Tropical and Public Health Institute, Basel, Switzerland; University of Basel, Basel, Switzerland; Department of Immunology, London School of Hygiene and Tropical Medicine, London, UK

**Keywords:** Malaria, Model, Serology, Measuring transmission intensity, Antibody titre, Cross-sectional data, Force of infection, *Plasmodium falciparum*

## Abstract

**Background:**

Serological data are increasingly being used to monitor malaria transmission intensity and have been demonstrated to be particularly useful in areas of low transmission where traditional measures such as EIR and parasite prevalence are limited. The seroconversion rate (SCR) is usually estimated using catalytic models in which the measured antibody levels are used to categorize individuals as seropositive or seronegative. One limitation of this approach is the requirement to impose a fixed cut-off to distinguish seropositive and negative individuals. Furthermore, the continuous variation in antibody levels is ignored thereby potentially reducing the precision of the estimate.

**Methods:**

An age-specific density model which mimics antibody acquisition and loss was developed to make full use of the information provided by serological measures of antibody levels. This was fitted to blood-stage antibody density data from 12 villages at varying transmission intensity in Northern Tanzania to estimate the exposure rate as an alternative measure of transmission intensity.

**Results:**

The results show a high correlation between the exposure rate estimates obtained and the estimated SCR obtained from a catalytic model (r = 0.95) and with two derived measures of EIR (r = 0.74 and r = 0.81). Estimates of exposure rate obtained with the density model were also more precise than those derived from catalytic models.

**Conclusion:**

This approach, if validated across different epidemiological settings, could be a useful alternative framework for quantifying transmission intensity, which makes more complete use of serological data.

## Background

Malaria remains a leading cause of morbidity and mortality worldwide [1], with heterogeneous levels of endemicity across the globe [2]. Measuring malaria transmission intensity is a key element of monitoring changes in transmission and assessing the impact of anti-malaria interventions. The reference standard historically used for reporting malaria transmission intensity is the entomological inoculation rate (EIR), defined as the number of infectious bites per person per year (ibppy), estimated by catching mosquitoes most commonly using light-traps and then dissecting these mosquitoes to estimate the sporozoite (infectious) rate. Despite its usefulness in providing a direct estimate of the force of infection, this method is time consuming, expensive and can lack precision, especially in low endemicity areas. The prevalence of individuals carrying the parasite—termed parasite prevalence (PrP)—is an alternative measurement of malaria transmission intensity. This can be, estimated in cross-sectional surveys using microscopy, rapid diagnostic test (RDT), or increasingly using PCR methods to detect infection in individuals. This approach is in widespread use, with population-level representative samples now undertaken in many malaria endemic areas as part of national Malaria Indicator Surveys [3]. However, although PrP can be estimated rapidly in populations, it is subject to seasonal variation (e.g. 30 % variation between the peak and trough in highly seasonal areas in West Africa [4]), is affected by anti-malarial treatment levels, requires highly skilled staff (for microscopy and PCR), and lacks precision in low endemicity settings.

Serological data, which measures antibody responses to one or more malaria specific antigens, offer an alternative means to estimate past exposure to malaria [5]. Such data were used historically during the Global Malaria Eradication Programme [6] and after [7–9] to monitor changes in transmission. There are several advantages to using serology in lower transmission settings where obtaining precise estimates of PrP can be prohibitively expensive. Serological assays such as ELISA are simple, quick and cheap to perform. Antibodies persist for months or years after infection, therefore, the effect of seasonality in transmission is smoothed out. Also, the longevity of antibodies means that seroprevalence remains sufficiently high in low transmission settings to obtain meaningful estimates with achievable sample sizes. Serological data are typically analysed using catalytic models to estimate the antibody seroconversion rate (SCR)—the rate at which seronegative individuals become seropositive—as a proxy for the force of infection [8, 9]. However, one limitation of this method is that it is necessary to distinguish seropositives from seronegatives using continuous measures of antibody levels. For malaria this is typically achieved using sera from European (i.e. unexposed) volunteers to define a cut-off. However, there may be underlying differences between the immune responses in these unexposed volunteers and those living in endemic countries. An alternative approach is to fit mixture models to the bi-modal distribution of antibody levels [12–14]. However, this approach can be problematic in highly endemic areas where a large proportion of the population are seropositive.

Here is presented a continuous model of the acquisition and loss of antibodies which can be fitted to individual-level data on measured antibody levels from cross sectional surveys. An advantage of this approach is that it takes into consideration the full information contained in measurement of antibodies levels rather than reducing the data to seropositive/seronegative status. Estimates of malaria transmission intensity are thus derived without the use of cut-offs and with better precision than estimates obtained with binary seroprevalence data. The utility of the approach is illustrated by fitting the model to data from a cross-sectional survey in Tanzania across areas with differing endemicity and compare the results to traditional measures of malaria transmission intensity as well as to estimates obtained from catalytic models.

## Methods

### Data

Two age-stratified cross-sectional surveys were conducted in Tanzania. Individuals aged 0–46 years of age were sampled from 12 villages across three altitudes transects in North Pare, South Pare and West Usambara. In each transect, villages were selected at high, medium and low altitude, reflecting a range in malaria transmission intensity [10]. As infants may present with maternal antibodies, only individuals between one and 46 years were included in the analysis. Previous publications have already described the study design and epidemiology of malaria in this area [15]. Ethical approval was obtained from the institutional review boards of the National Institute of Medical Research of Tanzania, Kilimanjaro Christian Medical Centre, and the London School of Hygiene and Tropical Medicine. Individuals’ sera were collected and antibodies to the asexual stage merozoite antigens, Merozoite Surface Protein (MSP-1_19_) and Apical Membrane Protein (AMA-1), determined using ELISA [16]. Only anti-MSP-1_19_ antibodies were considered for these analyses, as these are assumed to be more immunogenic than anti-AMA-1 antibodies [17–19]. Measurements were recorded as optical densities which were log-transformed prior to analysis with measurements below the limit of detection (LoD) assigned an approximate LoD of 0.01.

### Density model

#### Model specification

A mathematical model was developed to describe the dynamics of acquisition and loss of antibodies in the population. The model assumes that, following exposure to an infectious bite which occurs at rate λ, an individual’s antibody level is boosted by *δ*(*x*_*t*_) where *x*_*t*_ is the base-10 logarithm of antibody density. In the absence of exposure, antibodies are assumed to decay exponentially at a constant rate *ρ*. Let *y*(*x*, *t*) denote the proportion of the population with antibody level *x*at time *t* and *K*(x^*^, *x*) denote the probability that individuals with (log_10_) antibody level *x* at time *t* are boosted to level x^*^(x^*^ > *x*) on exposure to an infectious bite between *t* and *δt* with *K*(x^*^, *x*) = 0 if x^*^ ≤ *x* (∫_*x*_^∞^*K*(x^*^, *x*) dx^*^ = 1). Then the distribution of antibody levels in the population is given by:

$$\begin{aligned} \frac{\partial y(x,t)}{\partial t}& = \int_{0}^{\infty } {\lambda \left[ {K(x,x^{*} ),y(x^{*} ,t) - K(x^{*} ,x)y(x,t)} \right]} dx^{*}\\ &\quad + \rho \frac{\partial y(x,t)}{\partial x} \end{aligned}$$

With ∫*λK*(*x*, x^*^) *y*(x^*^) dx^*^ corresponding to all the individuals with lower antibody levels who get boosted to level *x*upon exposure. Similarly ∫*λK*(x^*^, *x*) *y* (*x*)dx^*^ corresponds to the individuals with antibody levels *x*who get boosted to a higher level upon exposure and $\rho \frac{\partial y(x)}{\partial x}$ corresponds to the individuals losing their antibodies.

The model was numerically approximated by a version in which the log_10_ antibody density variable, *x*, was discretized by dividing the range of the variable into *N* compartments each of width D, with *x*_*i*_ denoting the value of (log_10_) antibody density at the mid-point of antibody class *i*. The first class represents measurements below the LoD, *x*_min_. Here *N* = 51, with D = 0.052 and *x*_*min*_ = −2. The resulting discrete model describes the dynamics of the proportion of the population in each antibody density category *i,* denoted*y*_*i*_, and is defined by the following set of ordinary differential equations:

$$\frac{{dy_{i} }}{dt} = \lambda \sum\limits_{\begin{subarray}{l} j < i \\ i \ne 1 \end{subarray} } {k_{ij} y_{j} + \frac{\rho }{\varDelta }y_{j + 1} - \lambda \sum\limits_{\begin{subarray}{l} h > i \\ i \ne N \end{subarray} } {k_{hi} y_{i} - } } \frac{\rho }{\varDelta }y_{i} \begin{array}{*{20}c} {} & {} \\ \end{array} 1 \le i \le N$$

where *h, i, j* index the *N* antibody level classes. The rates of exposure and decay of antibodies, *λ* and *ρ*, are assumed to be independent of antibody density and age. The probability that following exposure, antibody levels are boosted to class *i* from class *j*, *k*_*ij*_, is distributed according to a discretized lognormal distribution:

$$k_{ij} = \left\{ \begin{array}{ll} 0 &\quad{{if }\quad}i < j\\ F(x_{i} + \varDelta /2 - x_{j} ;\delta (x_{j} ),S) - F(x_{i} - \varDelta /2 - x_{j} ;\delta (x_{j} ),S)&\quad{{if }\quad}j \le i < N \\ 1 - F\left( {x_{N - 1} + \varDelta /2 - x_{j} ;\delta (x_{j} ),S} \right) &\quad{{if }\quad}i = N \end{array} \right.$$

where *F*(*z*, *δ*(*x*), *S*)is the cumulative density function at point *z* of the lognormal distribution with mean *δ*(*x*) and standard deviation *S*. *δ*(*x*) is the mean boost size, a function of the current log_10_ antibody level, *x*, assumed to be given by:

$$\delta (x) = \left\{ \begin{array}{ll} ae^{-bx} &\quad {{if }\quad}x > x_{{\rm min} } \\ \eta &\quad {{otherwise}} \end{array} \right.$$

where *a, b* and *η* are parameters. This model assumes that exposure increases the log of antibody density by a decreasing amount as current density increases.

The model is run at equilibrium and constant malaria exposure over the years is assumed. As a result, age of individuals is considered as a proxy for time.

#### Parameter estimation

A Bayesian approach was used to estimate the model parameters, summarized in Table 1, by fitting the model to the data from the 12 villages simultaneously, allowing only the exposure, λ_v,_ to vary by village. The rate of decay of antibodies was fixed to 0.7 years^−1^. Using *θ* to denote the estimated parameter vector and *D* the data, the multinomial log-likelihood is given by:

**Table 1:** Model parameters and their maximum values

Parameter	Description	Maximum value	Estimates median (95 % CrI)
*λ*_v_	Rate of exposure for village *v*	100	See Table 2
*a*	Maximum antibody boost size on exposure	10	0.242 (0.237–0.246)
*b*	Slope of dependence of antibody boost on current log_10_ optical density	10	0.090 (0.035–0.121)
*s*	Standard deviation for boost size distribution	10	0.018 (0.006–0.032)
*η*	Mean boost for individuals with no current antibody	100	0.026 (0.025–0.029)

$$l = \log (P(\,D|\theta )) = \sum\limits_{v} {\sum\limits_{t \ne 0} {\sum\limits_{i} {n_{i,t,v} \log (} } } y_{i,t,v} )$$

Here *n*_*i*,*t*,*v*_ and *y*_*i*,*t*,*v*_ are, respectively, the observed number and predicted proportion of individuals in antibody category *i* in village *v* at age *t*. The differential equations were numerically integrated in C using the Runge–Kutta method [20]. MCMC methods (using a Metropolis–Hastings algorithm [21]) were used to calculate the posterior distribution of the parameters. As all parameters were strictly positive we used a log-normal random walk proposal density and assumed uniform priors on [0, *max*], where *max* was the maximum permitted value of each parameter as listed in Table 1. Two runs of 500,000 iterations were performed for each run of the MCMC algorithm with a burn-in period of 50,000 steps. Chain convergence was checked visually. The output was then recorded every 200 iterations to generate a sample from the posterior distribution. The standard deviation of the proposal distribution was tuned in order to achieve appropriate mixing of the chains and an acceptance rate close to 20 % [22].

#### Catalytic model

A comparison of the estimates with those obtained using a previously described catalytic model [23] was performed. In this simple model the proportion of individuals who are seropositive at age t is given by:

$$y_{c} (t) = \frac{{\lambda_{c} }}{{\lambda_{c} + \rho_{c} }}\left[ {1 - e^{{ - (\lambda_{c} + \rho_{c} )t}} } \right]$$

where *λ*_*C*_ is the mean annual rate of conversion from seronegative to seropositive and *ρ*_*C*_ the mean annual rate of reversion from seropositive to seronegative. It should be noted that (for similar decay rates, *r*) the SCR estimated with the catalytic model is expected to be smaller in absolute magnitude than the exposure rate calculated from the density model, as exposure in the density model may not always lead to an antibody boost sufficient to caused seroconversion by the criterion used in the catalytic model. A Bayesian MCMC approach as described above was used for parameter estimation. The model was fitted to all villages simultaneously, again allowing *λ*_*C*_ to vary by village but with the constraint of estimating a single value for*ρ*_*C*_ across all villages. Two methods were considered to define individual’s seropositivity. In the first a fixed cut-off value of antibody density of 0.5 was used based on data from non-exposed European sera [10]. In the second a mixture model was fitted to the antibody level distribution for all the village’s data combined across all age groups. The mixture model assumes that the population is composed of a subpopulation of seropositive individuals making up a proportion *p* of the whole population, and a seronegative subpopulation containing the rest of the population. Antibody levels of individuals in each sub-population are normally distributed with parameters (*μ*_1_, *σ*_1_) for the seronegative group and (*μ*_2_, *σ*_2_) for the seropositive group. The parameters of the mixture model were estimated using MCMC methods with the following likelihood:

$$L(\mu_{1} ,\sigma_{1} ,\mu_{2} ,\sigma_{2} ,p;X) = \mathop \varPi \nolimits_{i = 1}^{n} \{ (1 - p)\phi (X_{i} ,\mu_{1} ,\sigma_{1} ) + p\phi (X_{i} ,\mu_{2} ,\sigma_{2} )\}$$

where $X_{i}$ is the value of antibody levels *X*_*i*_ ∊ {*X*_1_, …, *X*_*n*_} and *φ*(*X*, *μ*, *σ*) represents the probability density function for an observation *X* from a Normal distribution $N$ with mean *μ* and standard deviation σ. So that the overall distribution of antibody levels is (1 − *p*) *N*(*μ*_1_, *σ*_1_^2^) + *pN*(*μ*_2_, *σ*_2_^2^). For comparison with studies that use fixed cutoff, analyses were also conducted in which individuals with an antibody level greater than *μ*_1_ + 3*σ*_1_ are defined as seropositive.

## Results

**Fig. 1:**
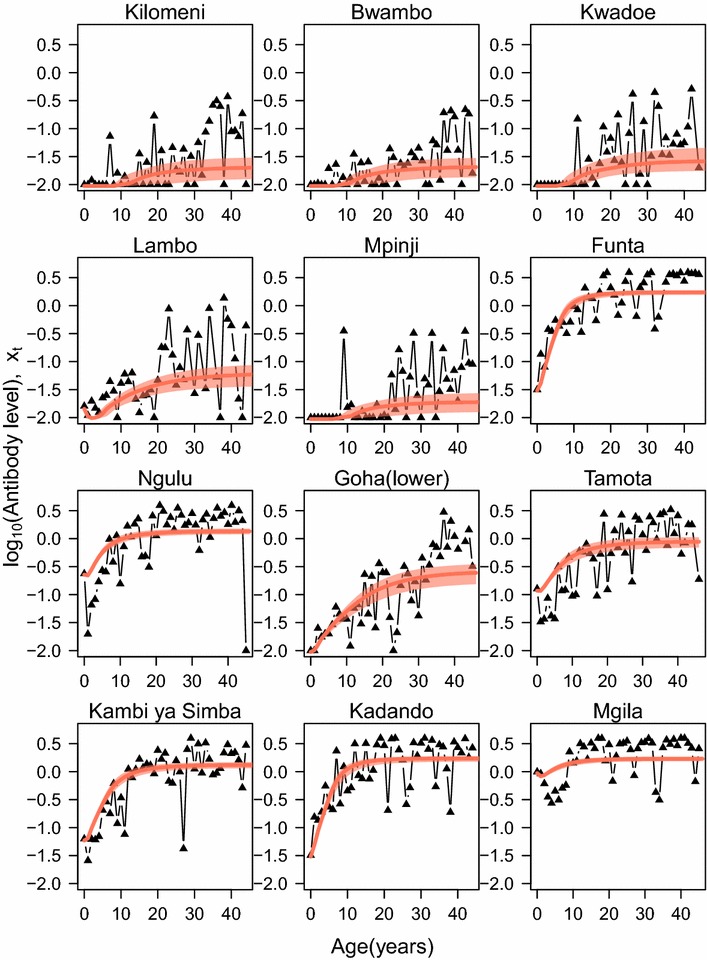
Antibody levels associated with age and village altitude for MSP-1_19_ antigens. *Black dots* indicate median estimates of antibody density for the actual data for each village, while median and 95 % credible intervals are represented in *red* for the model fit (*line* for median and *shaded area* for the credible interval). In each transect (each column), villages are presented with decreasing altitude/increasing transmission intensity from top to bottom

Data from 5227 individuals in the 12 villages were included in the analysis. The antibody levels of individuals in each village stratified by age are shown in Fig. 1. The overall trend shows an increase in mean antibody level in adults in the village with decreasing altitude and hence increasing transmission intensity in this setting [24–26]. As previously noted [10], two villages—Ngulu and Funta—have higher than expected antibody densities, suggesting higher transmission despite being at medium altitude. As expected, the trend is for antibody density to increase with age in each village, representing cumulative exposure to infection.

**Fig. 2:**
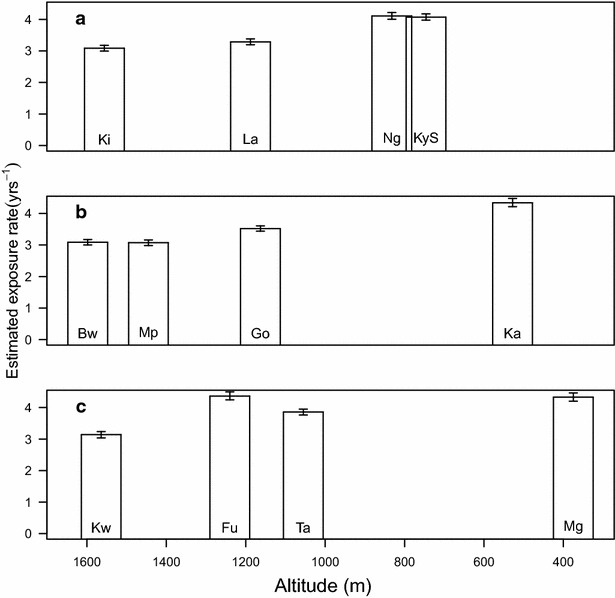
Estimated exposure rate (median ± 95 % CrI) by village. In each transect (presented by *boxes*) North Pare, (**a**), South Pare (**b**) and West Usambara, (**c**), the altitude for each village decreases from left to right and hence transmission intensity increases from left to right

The fitted density model is able to capture antibody density patterns across most of the villages (Fig. 1). In each transect, North Pare, South Pare and West Usambara, our estimate of the exposure rate increased with increasing transmission intensity (as indicated by decreasing altitude), with the exception, as expected, of Ngulu and Funta villages (Fig. 2).

**Fig. 3:**
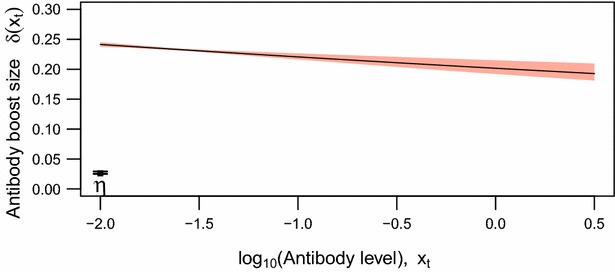
The estimated dependence of the mean antibody boost size (δ) on the exposed individual’s current log_10_ antibody level. Its 95 % Credible Interval is represented by the *shaded area*. *η* denotes the antibody boost size for individual lacking current circulating antibodies (Median and 95 % CrI)

The maximum boost size, *a*, was estimated to be 0.242 [95 % credible interval (CrI) 0.237–0.246] for individuals with antibody level above the detection threshold, and the slope of decline of boost with increasing current antibody level to be *b* = 0.09 (95 % CrI 0.035–0.121). As illustrated in Fig. 3, the low value of *b* means that the estimated antibody mean boost size declines approximately linearly with the current log_10_ antibody level. For the previously unexposed population, the mean boost size was estimated to be *η* = 0.026 (95 % CrI 0.025–0.029). The lack of overlapping CrIs for the estimates of *a* and *η* indicate that data allow us to distinguish the antibody responses of individuals who have never been exposed from those previously exposed who have low levels of antibodies.

**Fig. 4:**
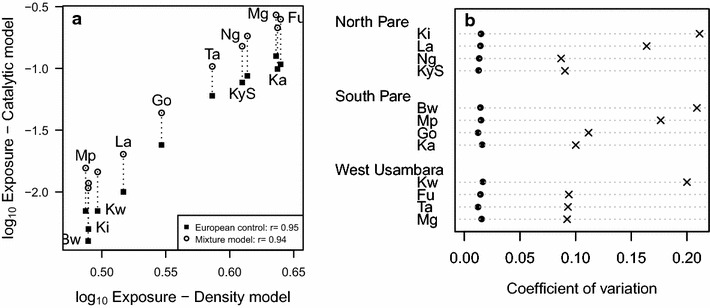
Comparison with seroconversion rate **a** Association of median estimates of exposure rates for different villages estimated using the antibody density model (*x-axis*) and the catalytic model (*y-axis*) using both European control (*squares*) and a mixture model to define the cut-off (*circles*). Note that the two measures are not equivalent but are highly correlated. **b** Plot of coefficient of variation (standard deviation of posterior/mean of posterior) of the exposure rate estimated from both the density model (*black circles*) and the catalytic model (*x*) using European controls. *Data* are presented for each village categorized by transect

A high correlation was observed between the estimates of exposure rates obtained using the density model, and that estimated by fitting a catalytic model to the data, using either European controls or a mixture model to derive the cut-off (see Fig. 4a). As anticipated, villages with high altitude (Bwambo, Kilomeni, Mpinji and Kwadoe) had lower estimates of exposure while villages with lower altitude (Kadando and Mgila) had higher estimates of exposure, and this was consistent for estimates obtained with both the density and catalytic models.

One of the advantages of estimating the exposure rate using the density model rather than SCR is that it makes fuller use of the continuous nature of the data, thus potentially increasing inferential power. The coefficient of variation of the exposure rate estimates, a measure of the precision of those estimates, is consistently smaller for the density model estimates than for the catalytic model estimates (Fig. 4b), demonstrating an increase in the precision of the estimate of transmission intensity obtained by fitting a density model rather than a catalytic model. This result is more marked for villages with lower transmission rates (higher altitude).

**Fig. 5:**
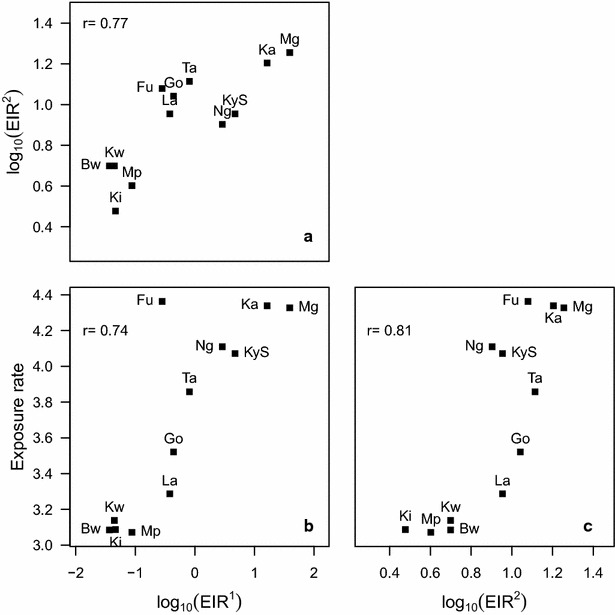
Comparison of metrics across villages. **a** EIR^1^ (calculated from altitude) with EIR^2^ (calculated from parasite prevalence); **b** exposure rate estimated with the antibody density model with EIR^1^; **c** exposure rate estimated with the antibody density model with EIR^2^. Speaman’s correlation coefficientis denoted by *r*

Figure 5 shows that the estimates of exposure rates were also highly correlated with the two different estimates of the EIR available for the study villages (and listed in Table 2).

**Table 2:** Summary of study data

Transect	Village	Altitude	N	PrP	EIR^1^	EIR^2^	Estimated λ_v_ median (95 % CrI)
North Pare	Kilomeni (Ki)	1556	411	1	0.047	0.08	3.087 (2.995–3.178)
	Lambo (La)	1188	355	10	0.38	1.11	3.287 (3.195–3.379)
	Ngulu (Ng)	832	486	8	2.9	0.84	4.109 (4.004–4.217)
	Kambi ya Simba (KyS)	746	494	10	4.7	1.11	4.071 (3.972–4.173)
South Pare	Bwanbo (Bw)	1598	485	3	0.037	0.26	3.085 (3.001–3.174)
	Mpinji (Mp)	1445	461	2	0.088	0.17	3.071 (2.983–3.158)
	Goha (Go)	1163	453	13	0.44	1.57	3.522 (3.437–3.607)
	Kadando (Ka)	528	382	25	16	4.45	4.339 (4.212–4.477)
West Usambara	Kwadoe (Kw)	1564	357	4	0.045	0.37	3.138 (3.037–3.236)
	Funta (Fu)	1240	429	17	0.28	2.38	4.364 (4.243–4.494)
	Tamota (Ta)	1055	449	19	0.81	2.74	3.857 (3.765–3.95)
	Mgila (Mg)	375	465	34	39	8.31	4.326 (4.198–4.463)

PrP denotes parasite prevalence measured with microscopy in children 0–4 years of age

EIR^1^ is estimated from altitude [EIR = 331.5 exp (−0.0057 × altitude) [10]]

EIR^2^ was estimated from the parasite prevalence data using a previously published relationship [39]

## Discussion

The utility of serological data to measure malaria transmission intensity has gained recognition in recent years [10, 11, 27] and is increasingly being incorporated in cross-sectional and longitudinal studies to monitor changes in transmission [28–32], identify “hotspots” of transmission [33–35] and to identify high risk groups [36]. One of the key advantages of the methods is their ease of use in the field, with new laboratory techniques enabling serological responses to multiple antigens to be made from dried blood spots that can be stored and transported without the need for refrigeration [37]. Classically the approach to analysing such data has been to distinguish seropositives from seronegatives using a cut-off value informed by unexposed European control populations [10, 11]. This has recognized limitations as the European control population may differ genetically in their immunological response to infection from the populations being analysed [38]. To avoid the need to incorporate a cut-off independent of the antibody background level, a density model was developed and fitted to serological data from a malaria endemic setting in Northern Tanzania.

The results demonstrate that estimates of the exposure rate obtained from fitting such models correlate highly both with previous estimates of the SCR obtained from the catalytic model as well as traditional measures of transmission intensity (the EIR) derived from altitude or from PrP data [10, 39]. Overall, the model fits the field data very well though less for older individuals in some areas, perhaps due to the small sample size in this age group. Simulation studies (not presented here) also showed a good fit to the data. The model, therefore, provides an additional method to estimate transmission intensity from serological data that avoids the need to determine a cut-off between seropositivity and seronegativity disregarding the background antibody level. One alternative approach to using European controls to define a cut-off has been to using a mixture model [12, 13]. Since this method can take into consideration the potential for misclassification of seropositive and seronegative individuals and in addition does not require an external dataset for standardization it provides an alternative appealing method for analysing serological data. However, one limitation of the mixture method approach is that it would not be appropriate in high transmission settings where the antibody distribution of seropositive and seronegative individuals becomes very difficult to distinguish. In contrast, the density model presented here performed equally well across all transmission settings.

An additional advantage of the density model over the catalytic model, as demonstrated here, is the improvement in the precision of the estimate of transmission intensity obtained by fitting a density model compared with that obtained from fitting a catalytic model. In particular, this was notably better in the lower transmission settings. This is not surprising, as by using a density model we use a greater degree of information in the data. However, it is also relevant from a practical perspective, as serological measures are likely to be of greatest use in areas of low transmission intensity where other commonly used measures (in particular PrP) lack precision. Thus by utilizing the full data set, this increased precision is likely to improve the ability to distinguish temporal and spatial trends in settings in which malaria has recently fallen to low levels [40, 41] as well as to monitor low-level transmission in countries working towards local elimination of the parasite [42, 43]. The ability to determine significant changes in transmission such as drops in antibody levels would be an important development for the evaluation of interventions [44, 45]. However, a counter argument to this is that the difference in precision may not be attributable solely to more effective use of continuous data but reflects inherent uncertainty about how exposure translates into prevalence, incorporated in the SCR but not in the exposure rate derived with the density model.

The point of comparison for the newly developed density model was chosen to be what is commonly used in practice, i.e. methods deriving thresholds to define seropositivity [10, 27, 46–48]. However, mixture models have long been used to also avoid arbitrary misclassification and directly derive seroprevalences and SCRs [12, 49–51]. Surprisingly these methods, despite being increasingly studied in the field of malaria [52] have not yet been adopted as common practices.

Additionally, note that the rate of seroconversion from the catalytic model and exposure rate from the density model measure different quantities. Indeed, the exposure rate measures the incidence of blood stage infections while the rate of seroconversion measures the rate at which individuals become seropositive, i.e. their antibody level increase above the cut-off value. One can, therefore, argue on the limited relevance in comparing these estimates. However it is their correlation with exposure measurements, which is of particular interest for comparing transmission intensity from different settings. Applying this approach to other serological datasets where additional measures of exposure are available is the obvious next step.

This model was developed using anti-MSP-1 antibodies as a proof-of-concept, as theses were assumed to be more immunogenic than other antibodies such as anti-AMA-1. However, such analysis could similarly be performed independently to other antibodies and the model could also be further developed to account for the dynamics of simultaneous antibodies.

Whilst the density model clearly has many advantages over the classical catalytic model, there are some limitations are worth noting. Firstly the density model outlined here, whilst biologically grounded, is a simplistic representation of the true process of antibody-acquisition and loss: it does not take into consideration more complex immune responses (such as interaction between antibody responses to different blood-stage antigens and/or between antibody- and cell-mediated immune responses [53]). Nevertheless, it would be interesting to check what effect, if any, incorporating these factors would have on the estimates of transmission as such data become available. Also, while providing a simplistic representation of the complex process, the model does not consider any age related changes in the affinity of the response [54, 55] nor in the age dependency of biting rates [56]. Whilst such aspects are clearly important, they cannot be estimated from cross-sectional data.

The process of loss of individual’s antibodies assumes a constant rate of decay of antibodies fixed to 0.7 years^−1^ corresponding to a half-life around 360 days. This value, which originated from a longitudinal study [57], made the assumption that antibodies detected for serological studies produced by long lived plasma cells. However, short-lived antibody responses to merozoite antigens are mostly observed [55, 58, 59] and by ignoring these, the model might over-estimate antibody levels. Sensitivity analyses on the rate of decay (not presented here) have shown a high correlation with exposure rate. As a result, the absolute value for the exposure might not be accurate depending on the assumption made for the rate of decay of antibodies but its usefulness in ranking settings according to their transmission intensity still remain.

Additionally, fitting a density model to serological data is computationally intensive and this may limit its wider utility. However, this can to some extent be overcome given the wider availability of statistical packages that can be used to perform such analyses and through sharing of code. To this end the R code used for this analysis is available from the authors on request. For wider use, the practicalities of the assay generating the serological data would need to include a strong component of standardization for the data between laboratories or conducted at different times to be compared confidently.

## Conclusion

In summary, the density model presented here provides a new method for analysing serological data that complements existing widely utilized tools for measuring malaria transmission intensity. No gold standard method has yet been developed for estimating exposure from serological data and this newly develop model represents an additional approach to do this. However, future development of this method is needed to test it against a wider set of transmission settings, incorporate methods for assessing spatial and temporal variations in exposure and to assess its utility in capturing changes in transmission following scaling up of malaria interventions.

## Abbreviations

SCR: seroconversion rate
PrP: parasite prevalence
EIR: entomological inoculation rate
CrI: credible interval
